# How low can you go? Changing the resolution of novel complex objects in visual working memory according to task demands

**DOI:** 10.3389/fpsyg.2014.00265

**Published:** 2014-03-28

**Authors:** Ayala S. Allon, Halely Balaban, Roy Luria

**Affiliations:** ^1^The School of Psychological Sciences, Tel-Aviv UniversityTel-Aviv, Israel; ^2^The Sagol School of Neuroscience, Tel-Aviv UniversityTel-Aviv, Israel

**Keywords:** visual working memory, capacity, resolution, electrophysiology, contralateral delayed activity

## Abstract

In three experiments we manipulated the resolution of novel complex objects in visual working memory (WM) by changing task demands. Previous studies that investigated the trade-off between quantity and resolution in visual WM yielded mixed results for simple familiar stimuli. We used the contralateral delay activity as an electrophysiological marker to directly track the deployment of visual WM resources while participants preformed a change-detection task. Across three experiments we presented the same novel complex items but changed the task demands. In Experiment 1 we induced a medium resolution task by using change trials in which a random polygon changed to a different type of polygon and replicated previous findings showing that novel complex objects are represented with higher resolution relative to simple familiar objects. In Experiment 2 we induced a low resolution task that required distinguishing between polygons and other types of stimulus categories, but we failed in finding a corresponding decrease in the resolution of the represented item. Finally, in Experiment 3 we induced a high resolution task that required discriminating between highly similar polygons with somewhat different contours. This time, we observed an increase in the item’s resolution. Our findings indicate that the resolution for novel complex objects can be increased but not decreased according to task demands, suggesting that minimal resolution is required in order to maintain these items in visual WM. These findings support studies claiming that capacity and resolution in visual WM reflect different mechanisms.

## INTRODUCTION

Visual working memory (WM) is a temporary buffer that can hold a limited amount of information in an active “on-line” state (Luck and Vogel, 2013). One primary characteristic of visual WM is its highly limited capacity. Many studies suggested that visual WM has an average capacity of about 3–4 objects (e.g., Luck and Vogel, 1997; Vogel and Machizawa, 2004; Xu and Chun, 2006; Awh et al., 2007). Nevertheless, there are robust individual differences in visual WM capacity and several studies have shown that these individual differences predict performance in a variety of aptitude measures. For example, high-capacity individuals get higher scores in fluid intelligence measures and in complex cognitive tasks such as verbal learning and problem solving compared to low-capacity individuals (Cowan et al., 2005; Fukuda et al., 2010; Johnson et al., 2013). Furthermore, impairments in visual WM functioning were associated with old age (Cashdollar et al., 2013) and with psychiatric disorders such as schizophrenia (Gold et al., 2003, 2006) and Alzheimer (Parra et al., 2010a,b, 2011). In addition, a recent electrophysiological study in monkeys (Buschman et al., 2011) suggested that each hemifield acts as an independent memory capacity limited resource (as limited-items models suggest; e.g., Zhang and Luck, 2008), and that within each hemifield memory resources are shared between the items represented in that hemifield (as flexible-resource models suggest; e.g., Bays and Husain, 2008). These findings indicate that visual WM plays an important role in guiding behavior, and therefore understanding how information is represented in visual WM constitutes a fundamental and important question in cognitive-neuroscience. Many studies tried to uncover the underlying processing mechanisms of visual WM and in particular the mechanism with which we represent information in visual WM using simple familiar objects such as color squares and arrows (e.g., Gao et al., 2009, 2011; Zhang and Luck, 2011; Machizawa et al., 2012; Murray et al., 2012). However, since we also encounter novel stimuli in our visual environment, it is also important to understand how we represent these novel items in visual WM. In the current study we tested how novel complex items are maintained in visual WM and whether it is possible to change the resolution with which these items are represented.

Several studies investigated the trade-off between the resolution (or quality) and the quantity of the items represented in visual WM, but failed to find such a trade-off. Zhang and Luck (2011) used the change-detection task (e.g., Luck and Vogel, 1997) in which participants are presented with a memory array (consisting of a set of items such as colors or orientations), followed by a retention interval (of about 1 s), and then a test array. Participants are instructed to indicate whether the test array is identical or different from the remembered memory array (e.g., when one of the objects changes its color). In Zhang and Luck (2011) study, the test array included a color wheel and participants reported the color of the test probe by clicking on the colors placed in the color wheel (for more details see Zhang and Luck, 2008). The precision (or resolution) needed to perform the task was manipulated by varying the number of distinct colors presented on the color wheel (e.g., nine colors in the low-precision condition and 180 colors in the high-precision condition). They assumed that if participants were able to trade precision with quantity, then more items would be remembered in the low-precision condition than in the high-precision condition. However, even after trying several manipulations intended to encourage a trade-off, no such trend was found, and Zhang and Luck (2011) concluded that participants were not able to increase the number of maintained items by storing them with low resolution. Importantly, they showed that when performance was based mainly on processes earlier than visual WM (i.e., iconic memory), the same manipulation was successful in causing a trade-off between resolution and quantity, so that participants were able to store more low resolution items in iconic memory. These findings indicate that the manipulation Zhang and Luck (2011) used was indeed valid, strengthening the conclusion that it is visual WM that is not sensitive to the trade-off between quantity and resolution.

Murray et al. (2012) also investigated this trade-off by manipulating the set-size and the precision in which simple stimuli (orientations) needed to be maintained in visual WM. They used a change-detection task in which participants had to decide in which direction the test probe changed (clockwise or anticlockwise; for the use of a similar task, see also Bays and Husain, 2008; Murray et al., 2011). Participants were informed in advance about the type of manipulation designed to induce the trade-off, so that either the magnitude of the change (e.g., the degree of the angular change), or the set-size, or both the magnitude of the change and the set-size were cued in advance. Murray et al. (2012) assumed that if a trade-off between quantity and resolution in visual WM is indeed possible, then performance for the same number of items (i.e., four items) should be better when anticipating a small rather than a large change, because the former encourages maintaining higher precision representations in visual WM. Murray et al. (2012) did not find any evidence for a better performance under conditions stressing quality (e.g., performance was similar when comparing four items in the small and large change conditions). Corroborating the findings by Zhang and Luck (2011), these results suggest that participants were not able to use preliminary information in order to bias the balance between the number of items and their resolution in visual WM.

Note that the studies reviewed above used accuracy as a primary measure to observe the trade-off between quantity and resolution in visual WM. Yet, it might be that a more sensitive measure to visual WM resource allocation is needed in order to observe this trade-off. Such measure is the contralateral delayed activity (CDA; Vogel and Machizawa, 2004). The CDA is an electrophysiological marker time locked to the onset of the memory array that enables to track the moment-by-moment deployment of visual WM resources exclusively during the maintenance stage. The CDA is a negative sustained wave at posterior scalp electrodes contralateral to the memorized side (Vogel and Machizawa, 2004; Vogel et al., 2005; McCollough et al., 2007). One important characteristic of the CDA is that its amplitude rises as a function of the number of items held in visual WM and not as a function of an increase in effort, reaching maximum amplitude in each individual’s maximum visual WM capacity (Vogel and Machizawa, 2004).

Several studies have tried to modulate the resolution for simple familiar items using a change-detection task while monitoring the CDA. Luria et al. (2010) investigated whether increasing the resolution of simple familiar items (i.e., color squares) will have an effect on the CDA as a “direct” measure of the visual WM capacity devoted to encode the items. In their Experiment 4, they compared the CDA between two conditions. In the low-resolution condition highly distinctive colors (i.e., blue, green, and yellow) were presented and in the high-resolution condition similar color hues were used (i.e., shades between blue and green). They assumed that if more visual WM capacity is needed in order to represent the colors in the high-resolution condition, then the CDA amplitude in this condition would be higher than the CDA amplitude for the low-resolution condition. However, Luria et al. (2010) found that the CDA amplitude for the high- resolution colors was similar to the CDA amplitude for low-resolution colors (and only changed as a function of the set-size). Their findings again suggest that participants were unable to increase the precision of representations in visual WM for simple familiar items.

Gao et al. (2011) also presented participants with simple items (arrows) and manipulated the set-size and the similarity between the memory and the test array while monitoring the CDA. They found that the CDA for two high-resolution arrows was the same as for two low-resolution arrows. Thus, similar to the findings of Luria et al. (2010), their findings suggest that participants were unable to increase the precision of representations in visual WM for simple familiar items.

Machizawa et al. (2012) used a similar procedure to the one used by Gao et al. (2011), but succeeded in showing an increase in resolution for simple familiar items. In their Experiment 2, Machizawa et al. (2012) presented participants with oriented bars and manipulated the resolution by modifying the similarity between the memory and the test array. They found that the CDA amplitude for two items in the high-resolution condition increased such that it was higher than the CDA of two low-resolution items. Contrary to previous studies, their findings suggest that participants were able to increase the precision of representations in visual WM for simple familiar items.

The studies reviewed above yielded mixed results concerning increasing the resolution in visual WM for simple familiar items while monitoring the CDA. Whereas Machizawa et al. (2012) succeeded in increasing the resolution, Gao et al. (2011) and Luria et al. (2010) did not find such an increase in the CDA amplitude. One possible explanation for Luria et al. (2010) results is that whereas Machizawa et al. (2012) used a spatial manipulation (i.e., the degree of angular change), Luria et al. (2010) used a change in color manipulation. Previous studies (e.g., Klauer and Zhao, 2004) suggested that visual WM and spatial WM operate under different components, which might explain the difference between the findings by Machizawa et al. (2012) and Luria et al. (2010).

Note that the studies reviewed above used simple familiar stimuli (e.g., arrows and colors). However, in the current study we tested whether the resolution of *novel complex* *objects* (e.g., random polygons) could be changed according to the task demands. Several previous findings (outlined below) showed that novel complex objects are maintained differently in visual WM, such that more visual WM resources are devoted in order to maintain them. Presumably, this extra capacity might reflect an increase in the object’s resolution. If this is indeed the case, we might be able to change this extra capacity according to the task demand, providing evidence that visual WM is sensitive to the resolution with which complex objects are maintained. We now turn to discuss the relevant studies arguing that visual WM devotes more resources when representing novel complex items.

Alvarez and Cavanagh (2004) presented participants with a variety of objects in a change-detection task and showed that as the object’s complexity increased, visual WM could hold fewer representations. For example, capacity estimates for random polygons and shaded cubes were smaller than capacity estimates for much more familiar simple objects such as colors or English letters. Their findings suggest that novel complex objects consume additional visual WM capacity resources, and thus resulting in fewer items that can be maintained.

Awh et al. (2007) challenged this conclusion by showing that poor performance (as evident in capacity estimates) in the change-detection task may be the result of a failure of the comparison process (i.e., the process that matches between the items held in visual WM and the test probe), rather than a failure during visual WM maintenance stage. Awh et al. (2007) manipulated the similarity between the memory- and the test-phase in a change-detection task with novel complex items (shaded cubes and Chinese characters) and familiar items (color squares). On change trials the test probe was either from a different category or from the same category as the item in the memory array, thus forming between-category and within-category change trials (e.g., in a between-category change trial a Chinese character could change to a shaded cube, but never to a different Chinese character, and in a within-category change trial a Chinese character could change to a different Chinese character, but never to a shaded cube). They demonstrated that for between-category change trials, capacity estimates for novel complex items were equivalent to capacity estimates for color squares, but for within-category change trials capacity estimates for novel complex items were lower than capacity estimates for colors. The findings of Awh et al. (2007) suggest that for novel complex items more errors arise during the comparison stage, therefore, resulting in an underestimation of their memory capacity. However, when the same items are presented with an easy comparison process, visual WM capacity estimation for novel complex objects is similar to that of simple familiar objects such as colors. In general, Awh et al. (2007) demonstrated that behavioral performance in the change-detection task does not always reflect the actual visual WM capacity.

One way to bypass the criticism raised by Awh et al. (2007) is to use the CDA, which as mentioned above is a direct electrophysiological index of the visual WM representation (Vogel and Machizawa, 2004). The CDA provides an index of the visual WM capacity that specifically reflects the number of objects maintained in visual WM. As opposed to accuracy measures that reflect the encoding, maintenance, and the comparison stage, the CDA is not limited by errors arising during the comparison stage of the change-detection task; because it is measured before the test array appears and therefore before the comparison process is initiated. Several previous studies used the CDA in order to track the deployment of visual WM resources to novel complex items (Gao et al., 2009, 2013; Luria et al., 2010).

Gao et al. (2013) employed a manipulation intended to increase the resolution for novel complex items. In their Experiment 1, Gao et al. (2013) used between- and within-category change blocks (as in the procedure used in Awh et al., 2007 study) with novel complex items (e.g., random polygons), in addition to blocks with simple familiar items (e.g., a triangle and a circle). Their results showed that for simple familiar items the CDA amplitude for four items was higher than that of two items, indicating a set-size effect. However, for complex novel items, in both the between- and within-category change conditions, the CDA amplitude for two items was similar to that of four items, indicating a lack of a set-size effect. Moreover, for novel complex items the CDA amplitude for the between-category (i.e., low-resolution) condition was similar to that of the within-category condition (i.e., high-resolution). Gao et al. (2013) claimed that since the low-resolution condition was similar to the high-resolution condition, detailed information (i.e., an increase in the resolution) was being stored in visual WM.

However, according to our view, in order to show an increase (or a decrease) in the resolution of novel complex items, one needs to directly compare the resolution of novel complex items to the resolution of simple items. Therefore, comparing the resolution of two novel complex items and four novel complex items is not sufficient in order to detect a change in the maintained resolution, because the lack of a set-size effect can result from a decrease in the CDA amplitude of four novel complex items rather than an increase in the CDA amplitude for two novel complex items. Thus, one can infer about an increased resolution for novel complex items by comparing it to simple items and demonstrating a difference. A close inspection of the results by Gao et al. (2013) shows that the CDA amplitude for two novel complex items (both in the high- and low-resolution conditions) was similar to that of two simple items. Therefore, their findings suggest that detailed information for novel complex items was not stored in visual WM, because the CDA amplitude of novel complex items and simple items remained the same.

In another study which also examined the representation of novel complex items, Luria et al. (2010) presented participants with blocks of random polygons and blocks of colored squares, and compared these conditions directly. They found that random polygons consumed more visual WM capacity as indicated by a higher CDA amplitude for two polygons compared to two colors. These findings suggest that novel complex items consume more WM capacity relative to simple objects such as colors. One possible interpretation of Luria et al. (2010) findings is that novel complex items are remembered as bits of information. Novel complex items such as random polygons used in previous studies do not have long-term memory traces. Hence, it might be that we break novel complex items into smaller recognizable pieces of information, and store these pieces in visual WM. Namely, we maintain novel complex items in a higher resolution (i.e., precision) than the resolution with which we maintain more familiar items such as colors, resulting in a higher CDA amplitude for random polygons.

**FIGURE 1:**
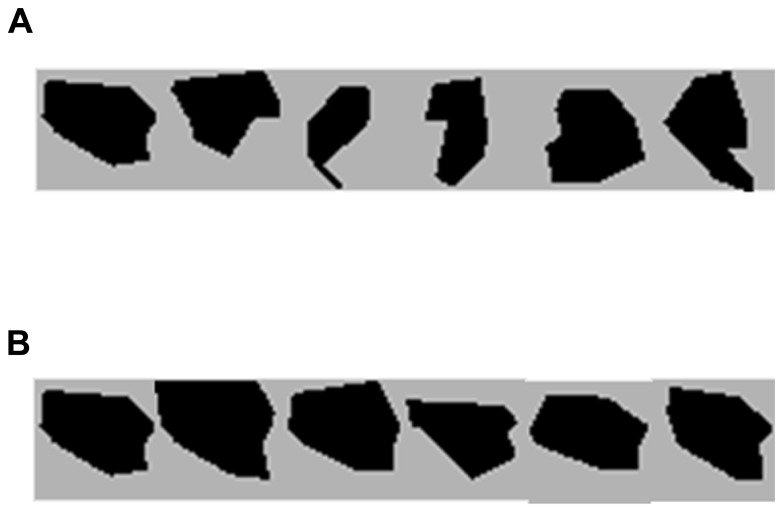
**(A)** Polygon prototype stimuli used in Experiments 1, 2, and 3. **(B)** An example of one of the six polygon-sets used in Experiments 1 and 3 with the prototype on the left side.

This interpretation has several interesting predictions. The main prediction is that the specific level of resolution with which a novel complex item is maintained in visual WM should be determined by the task demand. For example, a low-resolution task demand can require to distinguish between random polygons and other types of stimuli categories (such as a cube or a Chinese character). In this case, a polygon could be maintain at the category level, thereby decreasing its resolution, because only one piece of information is required in order to perform the task (i.e., only the category). A medium resolution task will require participants to distinguish between two completely different polygons (see **Figure 1A**). Now, an increased resolution is needed in order to discriminate between the polygons, so that additional pieces of information will be extracted in visual WM in order to maintain this task related resolution. Finally, a high-resolution task will require discrimination between very similar polygons with somewhat different angles (see **Figure 1B**). In this case, the precise contour of the stimulus needs to be maintained in order to detect a change, and presumably this will be achieved by “breaking” the polygon into even smaller pieces of information, and thus representing additional information in visual WM regarding each stimulus.

In the current study, across three experiments we modified the magnitude of change between the memory and the test array in a change-detection task. This manipulation changed the task demands (from low to medium to high, as was explained above), and therefore altered the level of resolution needed for successful performance. Importantly, we used the same set of stimuli across all three experiments and monitored the CDA. Specifically, we predicted that if the resolution for novel complex items such as random polygons can be changed, then the same item would consume a different amount of memory capacity according to the specific level of resolution induced by task demands.

In addition to polygon blocks, in each experiment we also presented blocks with simple colors that served as an identical baseline in all three experiments. This enabled us to compare the one polygon condition to the same one color and three colors conditions in each experiment in order to test whether the CDA amplitude changes according to task demands. In Experiment 1 we first replicated previous findings (Gao et al., 2009; Luria et al., 2010) by using polygon change trials in which a polygon always changed to a different polygon from a *different* *polygon-set* (see **Figure 1A**), requiring medium resolution in order to perform the task. In Experiment 2 we *decreased* the resolution by inducing a *between-category* change, so that a polygon always changed to a stimulus from a different category such as a cube (as in Awh et al., 2007). Note that novel items used by previous studies confounded novelty aspects of the stimulus with its complexity. However, by decreasing the task demands in Experiment 2 we eliminated the need to represent the item’s complexity (because only the category is important for the task), but at the same time maintained its novelty. Finally in Experiment 3, we *increased* the resolution that was required for successful performance (relative to Experiment 1) by inducing very small changes between the memory and the test phase using a different polygon from the *same polygon-set* (see **Figure 1B**).

## MATERIALS AND METHODS

### PARTICIPANTS

Thirty students from Tel-Aviv University (10 fresh participants in each experiment; 4 females, mean age of 24.6 years in Experiments 1; 4 females, mean age of 25.2 years in Experiment 2; 9 females, mean age of 23.8 years in Experiment 3) participated in a 180-min experimental session for either course credit or payment of 40 shekels per hour (approximately $10). All participants gave their informed consent after the procedures of a protocol approved by the Ethics Committee at Tel-Aviv University. All participants had normal or corrected-to-normal visual acuity and normal color-vision. One participant in Experiment 3 was replaced after reporting of having Synesthesia. Participants with more than 25% rejection rate due to eye-blinks or eye-movements were excluded from further analysis. None of the participants reached this limit.

### STIMULI AND PROCEDURE

We used the bi-lateral change-detection task (e.g., Vogel and Machizawa, 2004). Visual stimuli were displayed on a gray background on a 23-inch light emitting diode monitor with a 120 Hz refresh rate, using 1920 × 1080 resolution graphics mode.

In Experiment 1 stimuli were either color squares or random polygons. Color squares trials and polygon trials were presented in separate blocks in all three experiments because while for color squares the type of change on change trials was identical in all three experiments, the type of change for polygons was different in each experiment in order to manipulate the task demand. This manipulation enabled us to always use the color condition within the same task difficulty across experiments. From a viewing distance of approximately 60 cm, each color square subtended approximately 1.1° × 1.1° of visual angle, and each polygon subtended approximately 1.2° × 1.2° of visual angle. Color squares were drawn in six highly discriminable colors (yellow, green, cyan, blue, magenta, and red). Six prototypes of random black polygons and five additional variations for each prototype were generated using a custom Matlab (The Mathworks, Inc.) function (ShapeFamily; Collin and McMullen, 2002), for a total of six polygon-sets containing six members in each set (see **Figure 1B**). On each trial, the exact positions of the stimuli were randomized, with the constraint that the distance between the centers of each stimulus would be no less than 3° apart. Stimuli were randomly selected in the beginning of each trial, with the restrictions that the same number of stimuli appeared on each side of the fixation and that any color square or polygon could appear no more than once on each side of fixation.

**FIGURE 2:**
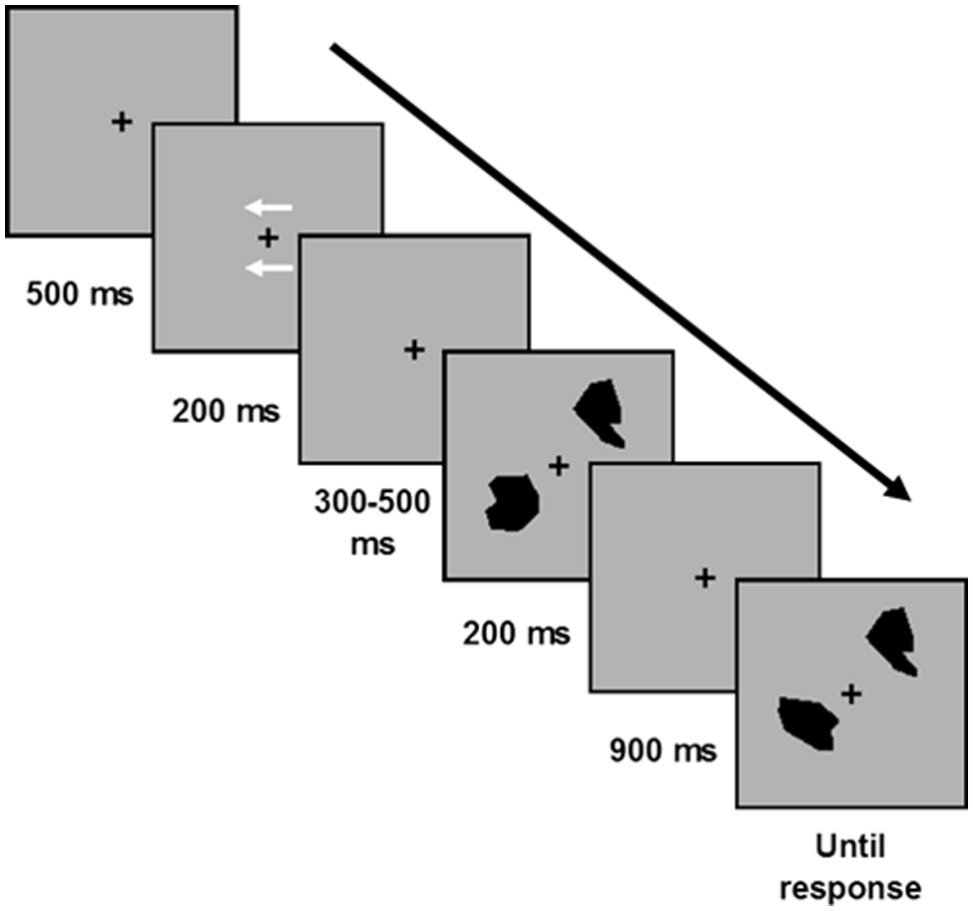
**The change detection paradigm.** Each trial began with a fixation cross (500 ms), followed by an arrow cue (presented for 200 ms) that indicate the relevant side for the upcoming trial. Then a memory array containing one or three polygons or one or three color squares on each side of fixation was presented for 200 ms (400 ms in Experiment 3); followed by a 900 ms retention interval and then a test probe in each side of fixation. Participants judged whether the test probe was the same or different as the item appeared in that location in the memory array.

Each trial started with a fixation cross (“+”) presented in the middle of the screen for 500 ms. Then, two arrow-cues were presented for 200 ms above and below the fixation, indicating the to-be-attended side for the upcoming memory array (right or left, with equal probabilities). Participants were instructed to memorize only the stimuli presented on the side indicated by the arrows. After a random interval (300, 400, or 500 ms from the cues offset), a memory array containing either the polygons or the colors was presented for 200 ms, followed by a retention interval of 900 ms (during which only the fixation cross was presented), and then a test probe (appearing at each side of the fixation) which remained on the screen until a response was emitted (see **Figure 2**). Participants made an unspeeded response via button press (“Z” and “/” keys on the computer keyboard, counterbalanced across participants) to indicate whether the test probe at the to-be-attended side was the same or different relative to the item in that location in the memory array (with equal probability for same and different trials; the test probe at the other side was always the same as the one in the memory array). On changed trials the test probe was a stimulus that was not already present in the memory array, but was always from the *same*-*category*. Namely on color blocks a color square was always replaced by a different color square, and on polygon blocks a polygon was always replaced by a different polygon from a *different polygon-set*.

As mentioned, color squares and polygons were presented in separate blocks, and in every trial the memory array contained either one or three stimuli (randomly determined with equal probabilities). Participants started with a practice polygon block of seven trials, followed by a practice color block of seven trials. There were 22 experimental polygon blocks and six experimental color blocks, each consisting of 40 trials. The blocks were presented in a fixed order: every fifth block was a color block, and the last block of the experiment was also a color block (following two, instead of four polygon blocks). This order was chosen to average practice and other order effects across conditions.

In Experiment 2, color blocks were identical to Experiment 1. However, this time instead of blocks with only polygon trials, we used mixed-categories blocks with stimuli from five different categories to create a between-category change following Awh et al. (2007) design. We used shaded cubes, polygons, Chinese characters, English letters, and color squares, and each category included six items for a total of 30 stimuli. For polygons and colors we used the same prototypes from Experiment 1. Shaded cubes and Chinese characters were adopted from Alvarez and Cavanagh (2004), and as English letters we used A, E, G, K, N, and R printed in black color. The shaded cubes subtended approximately 1.5° × 1.5° of visual angle, the Chinese characters subtended approximately 1.2° × 1.2° of visual angle, and the English letters subtended approximately 0.7° × 0.9° of visual angle. In the beginning of each trial stimuli were randomly selected, with the restrictions that a certain category (e.g., English letters) could appear only once on each side. On change trials, the test probe was always from a *different category* relative to the memory array (i.e., a polygon could change to a shaded cube, a Chinese character, an English letter, or a colored square, but never to a different polygon).

Experiment 3 was the same as Experiment 1, except as noted below. In the polygon blocks, six prototypes of random black polygons and five additional variations for each prototype were generated, for a total of six polygon sets containing six members in each polygon-set (see **Figure 1B** for an example of a polygon-set). The test probe on change trials was always a different member of the *same* *polygon-set*. We presented the memory array for 400 ms, instead of 200 ms. Previous findings (Luria et al., 2010, Experiment 3) indicated that prolonging the exposure time of the memory array increases the accuracy for detecting a polygon change, without affecting the CDA. Participants were informed that changes in polygon blocks would be from the same polygon-set and thus very subtle, and were encouraged to encode every aspect (i.e., angles) of the polygon.

### ELECTROENCEPHALOGRAPHY RECORDINGS

The electroencephalography (EEG) was recorded inside a shielded Faraday cage using a Biosemi Active Two EEG recording system (Biosemi B. V., Amsterdam, The Netherlands). Data was recorded from 64 scalp-electrodes at locations of the extended 10–20 system, as well as from two electrodes placed on the left and right mastoids. The horizontal electrooculogram (EOG) was recorded from electrodes placed 1 cm to the left and right of the external canthi to detect horizontal eye movement, and the vertical EOG was recorded from an electrode beneath the left eye to detect blinks and vertical eye movements. The single-ended voltage was recorded between each electrode site and a common mode sense electrode (CMS/DRL). Data was digitized at 256 Hz.

Offline signal processing and analysis was performed using EEGLAB Toolbox (Delorme and Makeig, 2004), ERPLAB Toolbox (erpinfo.org/erplab), and custom Matlab scripts. All EEG signals were referenced offline to the average of the left and right mastoids. The continuous data were segmented into epochs from -200 to +1100 ms relative to onset of the memory array in Experiments 1 and 2 and from -200 to +1300 ms relative to onset of the memory array in Experiment 3. Data was normalized relative to a 200 ms window before the onset of the memory array in all three experiments. Artifact detection was performed using a pick-to-pick analysis, based on a sliding window of 200 ms wide with a step of 100 ms. Trials containing activity exceeding 80 μV at the EOG electrodes or 100 μV at the analyzed electrodes (P7, P8, PO7, PO8, PO3, and PO4) were excluded from the averaged ERP waveforms. This procedure resulted in a mean rejection rate of 7% in Experiment 1, 6.4% in Experiment 2, and 6.4% in Experiment 3. The analysis included at least 80 trials per condition per subject in the color blocks in all three experiments, and at least 260 trials per condition per subject in the polygon (Experiments 1 and 3) or mixed-categories (Experiment 2) blocks. The epoched data was then averaged and low-pass filtered using a non-causal Butterworth filter (12 dB/oct) with a half-amplitude cutoff at 30 Hz. Only correct trials were included in the analysis.

### CDA ANALYSIS

Separate average waveforms for each condition were generated, and difference waves were constructed by subtracting the average activity recorded from the electrodes ipsilateral to the memorized array from the average activity recorded from electrodes contralateral to the memorized array. For statistical purposes, we used the average activity between 300 and 900 ms time locked to onset of the memory array. For the ease of description purposes, we will only present the results for the average of PO7/PO8, P7/P8, and PO3/PO4 electrodes. However, we also analyzed the results separately for each electrode pair and found similar patterns of activations.

## RESULTS

### EXPERIMENT 1

#### Behavioral

Accuracy levels for all three Experiments are presented in **Table 1**. The accuracy data revealed a decrease in accuracy as the set-size increased and this decrease was greater for polygons than for color squares. An analysis of variance (ANOVA) with stimulus-type (polygons, color squares) and set-size (1 and 3) as within-subject variables on accuracy levels as a dependent variable showed a significant main effect for stimulus-type, *F*(1,9) = 61.96, MSE = 0.00, *p* <0.001, $\eta_{p}^{2}$ = 0.87, and set-size, *F*(1,9) = 430.19, MSE = 0.00, *p* <0.001, $\eta_{p}^{2}$ = 0.97. The interaction for stimulus-type and set-size was also significant, *F*(1,9) = 49.56, MSE = 0.00, *p* <0.001, $\eta_{p}^{2}$ = 0.84. Planned comparisons showed that the decrease in accuracy between set-size one and three was significant for colors, *F*(1,9) = 70.97, MSE = 0.00, *p* <0.001, $\eta_{p}^{2}$ = 0.88, and for polygons, *F*(1,9) = 362.20, MSE = 0.00, *p* <0.001, $\eta_{p}^{2}$ = 0.97.

**Table 1:** Accuracy and standard deviation (in parenthesis) for Experiments 1, 2, and 3 across the different conditions.

One color	One polygon	Three colors	Three polygons
**Experiment 1**
0.96 (0.02)	0.92 (0.05)	0.87 (0.04)	0.71 (0.06)
**Experiment 2**
0.98 (0.02)	0.98 (0.01)	0.90 (0.06)	–
**Experiment 3**
0.99 (0.01)	0.75 (0.07)	0.91 (0.05)	0.58 (0.03)

#### Electrophysiology

We first wanted to make sure that there were no differences in the CDA amplitude between our color control conditions (i.e., one color and three colors) across the three experiments. An ANOVA with set-size (one color and three colors) as a within-subject variable and Experiment (Experiment 1, 2, and 3) as a between-subject variable on the CDA mean amplitude as a dependent variable revealed only a significant main effect for set-size, *F*(1,27) = 65.14, MSE = 0.34, *p* < 0.001, $\eta_{p}^{2}$ = 0.70, such that the CDA amplitude for three colors was higher than for one color, replicating previous findings (Vogel and Machizawa, 2004; Vogel et al., 2005; McCollough et al., 2007; Luria et al., 2010). Neither the main effect for Experiment, *F*(2,27) = 1.06, *p* > 0.3, $\eta_{p}^{2}$ = 0.07, nor the interaction between set-size and Experiment were significant (*F* < 1). Thus we were able to use the color conditions as control conditions across the three experiments.

**FIGURE 3:**
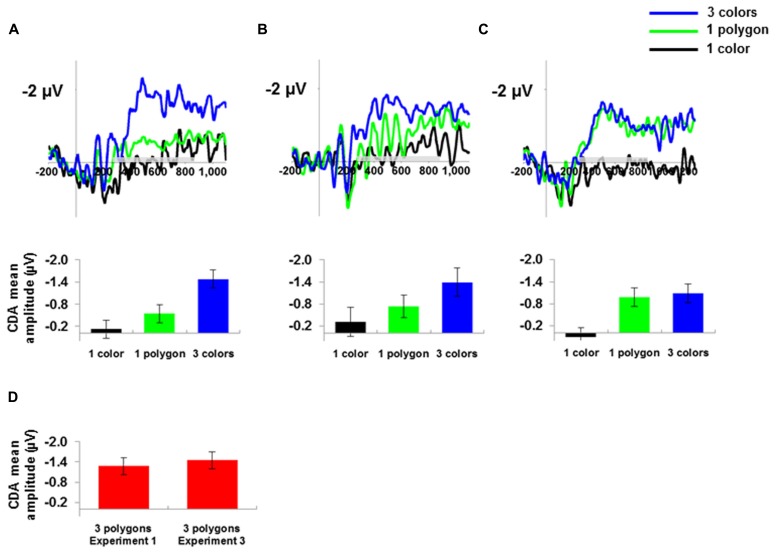
**The CDA wave (timed locked to the memory array) and mean amplitude in μV for one color, one polygon and three colors for **(A)** Experiment 1, **(B)** Experiment 2, and **(C)** Experiment 3.** The gray bar above the *x*-axis indicates the time range for calculating the mean amplitude. **(D)** The CDA mean amplitude in μV for the three polygons condition in Experiment 1 and 3. 95% confidence intervals were calculated according to Loftus and Masson (1994).

The CDA waveforms and mean CDA amplitude values for one color, one polygon, and three colors for all three experiments are presented in **Figures 3A–C**. The CDA mean amplitude for three polygons in Experiment 1 and 3 are presented in **Figure 3D**. An ANOVA with stimulus-type (polygons, color squares) and set-size (1 and 3) as within-subject variables with the CDA mean amplitude as a dependent variable showed a significant main effect for set-size, *F*(1,9) = 31.28, MSE = 0.35, *p* < 0.001, $\eta_{p}^{2}$ = 0.77. The main effect for stimulus-type was not significant, *F*(1,9) = 1.17, MSE = 0.10, *p* > 0.3, $\eta_{p}^{2}$ = 0.11. The interaction for stimulus-type and set-size was significant, *F*(1,9) = 8.03, MSE = 0.12, *p* <0.05, $\eta_{p}^{2}$ = 0.47, such that the set-size effect was larger for colors then for polygons. Planned comparisons confirmed this pattern and showed that the CDA amplitude for three colors was significantly higher than for one color, *F*(1,9) = 29.18, MSE = 0.31, *p* <0.001, $\eta_{p}^{2}$ = 0.76, indicating a set-size effect for simple objects (Vogel and Machizawa, 2004; Vogel et al., 2005; McCollough et al., 2007; Gao et al., 2009; Luria et al., 2010). In addition, we found a set-size effect for polygons such that the CDA amplitude for three polygons was significantly higher than for one polygon, *F*(1,9) = 17.57, MSE = 0.15, *p* <0.01, $\eta_{p}^{2}$ = 0.66, indicating that one polygon did not exhaust WM capacity.

We then compared the CDA amplitude for one color and one polygon. As can be seen in **Figure 3A**, planned comparisons showed that the CDA amplitude for one polygon was higher than for one color, *F*(1,9) = 13.54, MSE = 0.06, *p* < 0.01, $\eta_{p}^{2}$ = 0.6, replicating previous findings (Gao et al., 2009; Luria et al., 2010). Next we compared the CDA amplitude for one polygon and three colors in order to test whether our between-set manipulation for polygons increased the level of consumed memory capacity to be equivalent to the memory capacity of three colors. The CDA amplitude for three colors was significantly higher than for one polygon, *F*(1,9) = 23.47, MSE = 0.18, *p* < 0.001, $\eta_{p}^{2}$ = 0.72, indicating that one polygon consumed less memory capacity resources then three colors.

### EXPERIMENT 2

The results of Experiment 1 replicated previous findings (Gao et al., 2009; Luria et al., 2010). We demonstrated that one polygon consumed more visual WM capacity relative to one color, presumably because it was represented in a higher resolution. The purpose of Experiment 2 was to further test if the task demand can *decrease* the resolution with which polygons are represented. To this end, we used only *between-category* change trials, similar to the procedure used by Awh et al. (2007). As noted before, novel items such as random polygons are confounded with the complexity of the stimuli. However, by decreasing the task demands we eliminated the need to represent the item’s complexity (because only the category is important for the task), but at the same time maintained its novelty.

#### Behavioral

We first analyzed accuracy levels for each of the category-types in the one object trials condition. An ANOVA with category-type (polygons, Chinese characters, English letters, shaded cubes, colors) as a within-subject variable on accuracy levels as a dependent variable showed no difference between the various categories, *F* < 1, thus replicating previous findings (Awh et al., 2007) who showed that when the memory- and test-array similarity is low (by applying a *between-category* change), accuracy for different stimulus types is the same as for simple colors. Thus for further analysis accuracy in this condition was averaged across category-type. An additional ANOVA with stimulus-type (mixed-categories, colors) and set-size (1 and 3) as within-subject variables showed a significant main effect for set-size, *F*(1,9) = 23.53, MSE = 0.00, *p* < 0.001, $\eta_{p}^{2}$ = 0.72. The main effect for stimulus-type and the interaction for set-size and stimulus-type were not significant, *F* < 1.

#### Electrophysiology

In this experiment polygons were presented along with other stimulus types (e.g., shaded cubes and Chinese characters; mixed-categories condition) in order to form a *between-category* change, and reduce the resolution in which the items were maintained in visual WM. Note that a color was presented both as a control condition (in a separate color block containing only color squares) and in the mixed-categories condition together with the polygon and the other items. An ANOVA with stimulus-type (mixed-categories, colors) and set-size (1 and 3) as within-subject variables with the CDA amplitude as a dependent variable showed a significant main effect for set-size, *F*(1,9) = 19.93, MSE = 0.44, *p* < 0.01, $\eta_{p}^{2}$ = 0.68. The main effect for stimulus-type (*F* < 1) and the interaction for set-size and stimulus-type were not significant, *F*(1,9) = 2.95, MSE = 0.06, *p* > 0.1, $\eta_{p}^{2}$ = 0.24.

Since we wanted to test whether the task demand in the current experiment decreased the resolution in which one polygon was represented, in a separate analysis we analyzed the one polygon trials from the mixed-categories condition and compared it to the one color trials in the mixed-categories condition and to the one color trials in the control condition (i.e., blocks that involved only colors). As can be seen in **Figure 3B**, the CDA for one polygon was higher than the CDA for one color in the mixed-categories condition, *F*(1,9) = 6.97, MSE = 0.19, *p* <0.05, $\eta_{p}^{2}$ = 0.43, and in the control condition, *F*(1,9) = 3.73, MSE = 0.24, *p* = 0.08, $\eta_{p}^{2}$ = 0.29 (marginally significant). These results indicate that the polygon’s resolution was higher than the resolution of a color, even though the task required remembering all items at the category level and ignoring the specific details of the object. We also compared the CDA amplitude for one color in the control condition and one color in the mixed- categories condition. As expected, the CDA amplitude for one color in the control condition was similar to that of one color in the mixed-categories condition, *F* <1. We then compared the CDA amplitude for three colors in the control condition and one polygon in the mixed-categories condition. As in Experiment 1, the CDA amplitude for three colors was significantly higher than for one polygon, *F*(1,9) = 10.26, MSE = 0.2, *p* <0.05, $\eta_{p}^{2}$ = 0.53.

### EXPERIMENT 3

The evidence from Experiment 2 entails a few important notions. First, we behaviorally replicated previous findings by Awh et al. (2007) by showing that when the memory and test phase similarity is low, performance for novel complex items is equivalent to performance for familiar colors. However, we did not find the same findings when looking at the CDA amplitude. The CDA amplitude for one polygon was higher than for one color. Importantly, this difference suggests that the resolution for novel complex objects in visual WM cannot be further decreased even when the task allows representing the novel items at the category level without any requirement for maintaining specific details regarding each stimulus.

In Experiment 3 we sought to *increase* the resolution with which polygons are maintained in visual WM. In order to do so, we used six prototypes of random black polygons and five additional variations for each prototype, for a total of six polygon-sets containing six members in each polygon-set (see **Figure 1B** for an example of a polygon-set). On polygon change trials the polygon changed to a different polygon from the *same polygon-set*, and thus inducing very small changes between the memory and the test phase.

#### Behavioral

As in Experiment 1, accuracy data revealed a decrease in accuracy as the set-size increased and this decrease was greater for polygons than for color squares. An ANOVA with stimulus-type (polygons, color squares) and set-size (1 and 3) as within-subject variables on accuracy levels as a dependent variable showed a significant main effect for stimulus-type, *F*(1,9) = 287.97, MSE = 0.00, *p* <0.001, $\eta_{p}^{2}$ = 0.96, and set-size, *F*(1,9) = 239.71, MSE = 0.00, *p* <0.001, $\eta_{p}^{2}$ = 0.96. The interaction for stimulus-type and set-size was also significant, *F*(1,9) = 11.46, MSE = , *p* <0.01, $\eta_{p}^{2}$ = 0.56. Planned comparisons showed that the decrease in accuracy between set-size one and three was significant for colors, *F*(1,9) = 14.83, MSE = 0.00, *p* <0.01, $\eta_{p}^{2}$ = 0.62, and for polygons, *F*(1,9) = 192.70, MSE = 0.00, *p* <0.001, $\eta_{p}^{2}$ = 0.95.

#### Electrophysiology

An ANOVA that included the same variables as the accuracy analysis with the CDA amplitude as a dependent variable showed a significant main effect for stimulus-type, *F*(1,9) = 23.84, MSE = 0.22, *p* <0.001, $\eta_{p}^{2}$ = 0.72, and set-size, *F*(1,9) = 16.82, MSE = 0.41, *p* <0.01, $\eta_{p}^{2}$ = 0.65. The interaction for stimulus-type and set-size was also significant, *F*(1,9) = 10.72, MSE = 0.12, *p* <0.01, $\eta_{p}^{2}$ = 0.54. Planned comparisons showed that the CDA amplitude for three colors was significantly higher than for one color, *F*(1,9) = 19.93, MSE = 0.36, *p* <0.01, $\eta_{p}^{2}$ = 0.68, indicating a set-size effect as in Experiment 1. Also as in Experiment 1 we found a set-size effect for polygons such that the CDA amplitude for three polygons was significantly higher than for one polygon, *F*(1,9) = 6.21, MSE = 0.17, *p* <0.05, $\eta_{p}^{2}$ = 0.40.

We then compared the CDA amplitude for one color and one polygon. As can be seen in **Figure 3C**, planned comparisons showed that the CDA for one polygon was higher than for one color, *F*(1,9) = 28.85, MSE = 0.20, *p* <0.001, $\eta_{p}^{2}$ = 0.54. Next we compared the CDA amplitude for one polygon and three colors. As expected and as opposed to Experiment 1 and 2, the difference between the CDA amplitude for one polygon and three colors was not significant, *F* <1, suggesting that increasing the task demands resulted in a higher resolution representation of the polygon in visual WM.

### ADDITIONAL ACROSS EXPERIMENT ANALYSIS

A one-way ANOVA with Experiment (Experiment 1,2, and 3) as a between-subject variable on the CDA mean amplitude as a dependent variable in the one polygon condition was conducted to see whether the task demands modulated the one polygon condition between the three experiments. The difference between the one polygon condition in the three experiments was not significant, *F*(2,27) = 1.23, MSE = 0.40, *p* = 0.30, $\eta_{p}^{2}$ = 0.08.

In addition we conducted an ANOVA for the polygon conditions in Experiment 1 and 3 with Experiment (Experiment 1 and 3) as a between subject variable and set-size (1 and 3) as a within-subject variable on the CDA mean amplitude as a dependent variable. The ANOVA revealed a significant main effect for set-size, *F*(1,18) = 21.96, MSE = 0.16, *p* <0.001, $\eta_{p}^{2}$ = 0.54. The main effect for Experiment (*F* <1) and the interaction for Experiment and set-size were not significant, *F*(1,18) = 1.10, MSE = 0.16, *p* = 0.30, $\eta_{p}^{2}$ = 0.05.

We should expect that the CDA for the one polygon condition would be significantly different between the three experiments and that the interaction for polygons between Experiment and set-size will also be significant. However, since these were between experiments comparisons with only ten participants in each experiment, they are likely to lack statistical power relative to the within-subject comparisons we performed.

## GENERAL DISCUSSION

The purpose of the current study was to investigate whether task demands can change the resolution of novel complex objects within visual WM. Whereas previous studies (Gao et al., 2009, 2011; Luria et al., 2010; Zhang and Luck, 2011; Machizawa et al., 2012; Murray et al., 2012) tested the resolution of simple familiar items, in the current study we tested the resolution for representing novel complex objects by maintaining the same quantity (i.e., one polygon) and trying to change the quality of that representation. Participants knew in advance about the type of change they will encounter, and were encouraged to maintain the items at a level that would lead to successful performance. In Experiment 1, we presented participants with novel polygons that could change to a polygon from a *different polygon-set*. In Experiment 2, we decreased the level of representation needed by inducing a *between-category* change (e.g., a polygon changing to a shaded cube but never to a different polygon). In Experiment 3, a polygon changed to a different polygon from the *same polygon-set* (resulting in a very subtle change in the polygon’s contour), which increased the level of representation needed to perform the task relative to Experiment 1. Importantly, the same sets of polygons were presented in all three experiments (in Experiment 2 we used only the prototypes of each polygon-set). In addition, we presented simple colors in separate blocks that served as an identical control condition across experiments.

In order to track the resolution of the maintained stimuli in visual WM, we monitored the CDA amplitude as an electrophysiological marker of allocating visual WM resources during the retention interval. Thus, our CDA results do not depend on the comparison process, shown to bias behavioral performance when complex information is employed in the change-detection paradigm (Awh et al., 2007).

The results of Experiments 1, 2, and 3 showed that the resolution for novel complex items could be increased but not decreased by the task demands. In Experiment 1 we replicated previous findings by Gao et al. (2009) and Luria et al. (2010). We demonstrated that the CDA amplitude for one polygon was higher than for one color but lower than for three colors. In Experiment 2, we decreased the task demands in order to investigate whether a polygon will be represented at a low resolution. However, the CDA amplitude for one polygon was still higher than the CDA amplitude for one color. These findings indicate that at least for polygons, the resolution for maintaining novel complex items in visual WM cannot be lowered beyond a certain limit. One possible interpretation for this result is that in 200 ms of exposure time participants already extracted detailed information into visual WM (Gao et al., 2013), which is why the CDA amplitude for one polygon was higher than for one color. Finally, in Experiment 3 the task required monitoring very small changes in the polygon’s contour, and indeed polygons were maintained in a higher resolution relative to Experiment 1, as indicated by the CDA amplitude for one polygon that was now similar to the amplitude of three colors.

Originally, the question of whether visual WM can trade quantity for quality was raised in light of the debate concerning the nature of visual WM capacity allocation. There are two classes of theories that explain how capacity is divided between the items that are held in visual WM. *Flexible-resource theories* claim that memory capacity is allocated in a flexible manner (Bays and Husain, 2008). Meaning, that as more resources are dedicated to an item, the more precise this item will be represented in visual WM. Conversely, when resources are allocated between several items, the precision of their representations will decrease. In contrast, according to *limited-item theories,* visual WM capacity is organized as a fixed number of slots rather than a pool of resources, so that capacity is divided by allocating the slots to the remembered objects (Zhang and Luck, 2008; Anderson et al., 2011).

*Flexible-resource theories* can naturally explain a trade-off between resolution and the number of maintained items because this is the nature of memory capacity resources if they are indeed “flexible.” In contrast, according to *limited-item theories* once the memory capacity is exhausted, visual WM cannot trade quantity for quality, because once a slot is not allocated to a specific object, this object should be completely left out of visual WM. Our results constrain *flexible-resource theories* since we were able only to increase the resolution for maintaining novel items in visual WM (Experiment 3), but not do decrease it (Experiment 2). We further argue that our results can be easily accounted for also by *limited-item theories*. First, Note that we changed the resolution of only one item, and even though it was complex, one polygon did not exhaust the entire WM capacity (as indicated by a significant increase in the CDA amplitude for three polygons relative to the CDA amplitude for one polygon in Experiments 1 and 3). This means that the increase in visual WM capacity for one polygon relative to one color observed in Experiment 1 may be the result of multiple slots devoted to representing this object. In addition, the further increase in the resolution for one polygon observed in Experiment 3, could simply reflect that visual WM allocated even more slots in order to maintain that item (about three slots as implied by the CDA amplitude which was similar between the one polygon conditions and three colors). Thus, the finding of the current research could be accounted for by both *limited-item theories* and* flexible-resource theories*. In addition, it might be interesting for future monkey electrophysiology studies to test the results of the current study in light of Buschman et al. (2011) account and see if the modulation of representation for novel complex items is the same as for simple items when manipulating the set-size between the two hemifields.

Furthermore, the findings of the present study support studies suggesting that capacity and the resolution in visual WM rely on different mechanisms (Xu and Chun, 2006; Fukuda et al., 2010). First, we were able to manipulate resolution independently from the number of maintained items (comparing Experiment 1 and 3). Second, the resolution in visual WM for novel complex items could not be decreased beyond a certain limit (Experiment 2). This finding suggests that although accuracy performance for novel complex objects was as good as for simple colors, visual WM still devoted more capacity resources in order to represent polygons. Thus, novel complex objects require minimal resolution for maintenance in visual WM.

## Conflict of Interest Statement

The authors declare that the research was conducted in the absence of any commercial or financial relationships that could be construed as a potential conflict of interest.
